# Learning a new geometric concept: The role of working memory and of domain‐specific abilities

**DOI:** 10.1111/bjep.12434

**Published:** 2021-06-19

**Authors:** Carlotta Rivella, Cesare Cornoldi, Sara Caviola, David Giofrè

**Affiliations:** ^1^ Department of Educational Sciences University of Genoa Italy; ^2^ Department of General Psychology University of Padua Italy; ^3^ Department of Developmental and Social Psychology University of Padua Italy; ^4^ School of Psychology University of Leeds UK

**Keywords:** fourth graders, geometrical learning, geometry, manipulatives, working memory

## Abstract

It has been suggested that not only domain‐specific factors but also working memory (WM) may play a crucial role in mathematical learning included Geometry, but the issue has not been deeply explored. In the present study, we examined the role of domain‐specific factors and of verbal versus visuospatial WM on geometric learning of a new geometrical figure (trapezoid), never presented previously by the teachers participating to the study, after a lecture also involving manipulatives. Results on 105 children in their Year 4 indicated that not only some domain‐specific components (geometric declarative knowledge and calculation) but also visuospatial working memory had a significant specific impact on the ability of solving geometric problems requiring to calculate the perimeter and the area of the new figure. On the contrary, verbal WM and geometrical mental imagery did not offer a specific contribution. These findings could have important educational implications, stressing the importance of taking into account the main different aspects supporting the acquisition of geometry.

## Background

Geometry enables individuals to make sense of the world by understanding geometric shapes, principles, and relationships (National Council for Teachers of Mathematics, 2000). Geometrical knowledge is indeed considered one of the most important areas of mathematics (Arcavi, 2003; Zhang, Ding, Stegall, & Mo, 2012). Therefore, understanding how children learn to master geometrical concepts, in order to be able to solve concrete and progressively more abstract problems, is of a vital importance in the education domain (Clements & Sarama, 2011). In the present study, we examined the issue by considering the acquisition of a geometrical concept that was completely new to the students, analysing what cognitive underpinnings come to play in a traditional instruction setting where manipulatives had also been introduced.

### Cognitive underpinnings of geometry

Learning experiences lead pupils to further their geometrical learning through the progressive acquisition of procedural and conceptual knowledge (Clements & Battista, 1992; Fisher, Hirsh‐Pasek, Newcombe, & Golinkoff, 2013; Piaget & Inhelder, 1967). Many authors highlighted the combined role of domain‐specific and domain‐general abilities in predicting academic achievement (see Caviola, Mammarella, Lucangeli, & Cornoldi, 2014; Fuchs, Geary, Fuchs, Compton, & Hamlett, 2016). Despite this, little progress has been made in furthering the understanding of cognitive processes, both domain‐specific and domain‐general, underpinning geometry, and in fact, these aspects have been scarcely investigated by the current literature (see Bizzaro, Giofrè, Girelli, & Cornoldi, 2018; Giofrè, Mammarella, & Cornoldi, 2014; Giofrè, Mammarella, Ronconi, & Cornoldi, 2013).

Domain‐specific abilities (McNeill, & Krajcik, 2009) refer to abilities, concepts, and knowledge that individuals develop in single domains such as mathematics, but not in other domains, for example, reading comprehension. In the acquisition of geometry, an important set of domain‐specific abilities is related to the geometrical terms defining geometrical basic concepts. The declarative knowledge of the appropriate vocabulary is understandably implicated in geometry that, on the surface, appears less language based (Spelke & Tsivkin, 2001; Vukovic & Lesaux, 2013). Children in primary schools are required to learn names and properties of a wide range of two‐ and three‐dimensional shapes and figures (Fisher et al., 2013; Mammarella, Todeschini, Englaro, Lucangeli, & Cornoldi, 2012; van Hiele, 1986) and an adequate knowledge of the basic concepts and terms is therefore needed in the acquisition of complex geometrical concepts (Bizzaro et al., 2018; Swindal, 2000). Furthermore, as geometry is strictly connected to data manipulation and measurement, a second set of domain‐specific abilities is represented by calculation skills (Mammarella, Giofrè, & Caviola, 2017), required, for example, to calculate perimeters and area of geometric figures. Finally, spatial geometrical abilities (Battista, 1990; Bizzaro et al., 2018; Clements, & Battista, 1992; Weckbacher & Okamoto, 2014), and geometrical mental imagery, that is the ability to mentally manipulate two‐ or three‐dimensional figures, seems to be crucially involved. The visuospatial representation of the problem has been shown to be the most effective strategy to solve a geometric problem compared to the memorization and application of an algorithm (Delgado & Prieto, 2004; McGuinness, 1993). However, it is unclear to what extent the eventual contribution of domain‐specific components, and in particular of spatial mental imagery, is due to working memory (WM) that supports them (Cornoldi & Vecchi, 2003; Meneghetti, Cardillo, Mammarella, Caviola, & Borella, 2017).

Domain‐general abilities refer to general cognitive skills that can be used across domains (McNeill & Krajcik, 2009). Working memory (WM), that is, the ability to temporarily maintain and manipulate information (Baddeley & Hitch, 1974) seems to be involved in a series of different domains including geometry, mathematics, or literacy (Alloway & Alloway, 2010). In the domain of geometry, WM seems to play an important role as solving geometric problems requires to maintain and manipulate visual and verbal materials (geometric figures, specific vocabulary, data, etc.; Mammarella et al., 2017). According to the tripartite model proposed by Baddeley and Hitch (1974), WM is articulated in two independent domain‐specific components (see also Baddeley, 1986; Engle, Kane, & Tuholski, 1999), respectively, processing verbal and visuospatial information, coordinated by a domain‐general component, the central executive. A large body of research has shown that both verbal and visuospatial WM predict the success in different mathematic domains (Bull & Scerif, 2001; Caviola, Mammarella, Cornoldi, & Lucangeli, 2012; Caviola et al., 2014; Mammarella, Caviola, Cornoldi, & Lucangeli, 2013), including geometry (Bizzaro et al., 2018; Giofrè et al., 2014).

The few studies that examined the relationship between WM and geometric achievement focused on the role of the visuospatial component of WM (VS‐WM) in geometric problem‐solving tasks (Giofrè et al., 2013; Mammarella et al., 2013; Zhang, 2017). According to these studies, higher VS‐WM seems to be related to higher scores in geometric tasks in both primary and secondary school children. However, in this area of research, even though geometry mainly involves visuospatial material, the verbal component of WM (V‐WM) has also resulted to be related to the geometric performance (Giofrè et al., 2014). This result could be due to the importance of definitions, mnemonic rules, verbal texts of problems in geometry, but also to the fact that V‐WM involves storage and processing resources that are shared with VS‐WM. Research is therefore needed in order to examine the specific contribution of V‐WM and of VS‐WM especially with reference to a well‐defined case of geometrical learning. In fact, previous studies concerning the relationship between WM and geometry has mainly used achievement general measures, involving heterogeneous aspects of geometrical learning (Bizzaro et al., 2018; Giofrè et al., 2013, 2014; Kyttälä, & Lehto, 2008) without considering what happens when new specific geometrical concepts are acquired via instruction.

### Instructional factors

Research on cognitive factors affecting geometrical learning must consider that instructional factors may also affect learning. Given its importance, geometry is included among the core standards in mathematics curricula worldwide, but substantial differences emerge among countries. Cross‐national studies have consistently shown that differences in students’ achievement are reflecting, at least in part, differences among curricula (Cai, 1995; Jackson, 1992; Schmidt, Houang, & Cogan, 2002). For example, while some nations introduce and integrate geometry very early in the curriculum (i.e., making geometry an integral part of education from kindergarten through junior high), other countries tend to delay geometry up until secondary school (Miyakawa, 2017).

School curricula are meant to provide a set of guides anchored to the expected abilities of students during different school Years, but differences may emerge also within a country in relation to instructions and teaching methods and quality (Fan, Trouche, Qi, Rezat, & Visnovska, 2018; Jones, Bokhove, Howson, & Fan, 2014). In particular, differences exist in the way content standards are implemented from curricula to instructions. Therefore, the variability in studies considering cognitive variables affecting geometrical learning may be reduced by focusing on a specific type of instructional strategy that appears particularly effective and could be the preferred one. Typical classroom instruction (i.e., direct instruction) is not often sufficient to reach all students, particularly in subjects like geometry where the content sequentially builds on and becomes increasingly layered (Clements, 2008; Clements & Sarama, 2011; Fuchs et al., 2015). Direct instruction mainly involves abstract representation of problems and their solutions, and thus, alternative instructional strategies may facilitate the acquisition, maintenance, and generalization of more complex skills. One way to accomplish this task is through the inclusion of manipulative materials (i.e., concrete materials that students arrange to represent a variety of mathematic relationships [Baki, Kosa, & Guven, 2011; Satsangi & Bouck, 2015]). One particularly useful, efficient, and effective strategy employing various manipulatives, in conjunction with direct instruction, is the so‐called concrete–semiconcrete–abstract (CSA) strategy (Yim & Hong, 2016).

Research has proven the efficacy of the CSA in teaching elementary‐level range of maths competences (Jordan, Miller, & Mercer, 1999; Miller & Mercer, 1993; Yim & Hong, 2016), including the area and perimeter (Cass, Cates, Smith, & Jackson, 2003). The efficacy of the CSA has been demonstrated in a wide range of math’s concepts (i.e., place value, addictions, subtractions and divisions facts, and algebra problem‐solving), particularly in students with learning disabilities (Maccini & Hughes, 2000; Miller, Mercer, & Dillon, 1992; Peterson, Mercer, & O'Shea, 1988), but the case of geometrical learning has not been deeply considered.

### The present study

The main aim of this study was to investigate the role of cognitive abilities on the acquisition of advanced geometrical knowledge. Differently from previous studies − in which geometric achievement was evaluated using declarative knowledge tasks (Giofrè et al., 2013) or using questions concerning a series of basic geometric figures that the child has acquired through many sources (Bizzaro et al., 2018) − in this study, we examined geometric learning through problem‐solving tasks. Our goal was to specifically evaluate the learning degree of a complex figure, such as the trapezoid, presumably new for all children, as teachers were previously invited to avoid referring to this figure. We considered the case of Italian children learning how to calculate the area and perimeter of the trapezoid, following an instruction session (involving manipulatives) that was identical for all the classes included in the study.

The Italian curriculum supports the sequential nature of geometrical learning, as described in the van Hiele’s (1986) levels of the development of geometric ability (Clements & Battista, 1992), and the formal introduction of concepts such as perimeter or area, and their calculation is introduced typically from Year 4, that is the focus of our study. The formal instruction on perimeter and area begins with figures such as square, rectangle, and triangle, while more complex and presumably less intuitive, figures (e.g., rhomboid or trapezoid) are introduced later on in the curriculum, when children have practiced with basic figures (e.g., triangles), and have automatized some basic procedures (e.g., calculating the area or the perimeter). Usually, the introduction of the trapezoid is not anticipated by previous information and suggestions concerning this figure and therefore can be a good case for examining how new geometrical knowledge is acquired.

There is an open debate involving educators and researchers on how curriculum contents should be taught to the primary school children (Fisher, Hirsh‐Pasek, & Golinkoff, 2012). However, there is some evidence suggesting that child‐centred and playful learning programmes, could promote sustained academic performance, as compared to more traditional, academically focused programmes (Diamond, Barnett, Thomas, & Munro, 2007; Fisher et al., 2013) and CSA teaching sequences for geometry, requiring the active manipulation of geometric figures, are becoming popular in primary schools of Italy and of many other countries. To examine factors underlying the acquisition of a new geometrical concept after an instruction session common to all the classes involved in the study, we decided that the teaching session included CSA. In the study, the teaching session was followed by a learning measure represented by a series of problems requiring to calculate either the perimeter or the area of trapezoids. To verify whether the information acquired was relatively stable and the measure in this way collected reflected a real learning, we decided to test children twice: immediately after the lecture and after two weeks from the lecture, with the request to the teachers to avoid activities concerning the trapezoid during this period.

Regarding the involvement of domain‐specific and general abilities, we decided to focus on (and preliminarily assessed) a series of variables that previous research had shown to be related to geometrical learning. Concerning domain‐general abilities, we considered the fact that the most cited ability is WM and we decided to focus on both visuospatial and verbal WM, taking into account the fact that both these components are highly articulated and different measures may define partly peculiar aspects of WM. In particular, in the selection of the tasks, we considered the proposal of Cornoldi and Vecchi (2003; see Cornoldi & Giofrè, 2014 for a review) that, both for verbal and visuospatial WM, a simple span task, a backward span task and an active task, requiring the selective recall of only a part of the presented information, may well represent different aspects of WM.

Concerning domain‐specific abilities, we also considered the aspects that have been more frequently associated with geometrical learning, that is, geometrical mental imagery, knowledge of geometric terms and basic concepts (geometrical declarative knowledge), and calculation. As we were mainly interested in examining the role of basic domain‐specific mathematical abilities, we decided to exclude the consideration of more specific skills concerning the abilities to calculate perimeter and area of other geometric figures that were presumably highly associated with our trapezoid learning measures.

Based on previous studies, we predicted that all these variables could be related to geometrical learning and that, due to the relationships existing between these variables, their unique contribution could be made evident when they were considered altogether, and the weight of each of them could be taken into account and then partialized.

## Methods

### Participants

The study involved 122 children in Year 4 and their teachers of mathematics. The children were attending six different classes in the town of Genova and Verona, northern Italy, and the math teacher of each class was involved in the study. Children were tested over the course of four assessment sessions. Children who were absent in one or more assessment sections were excluded from the analyses. The final sample consisted of 105 children (*M*_age_ = 9.63, *SD* = 0.32; 53 females). After school administration’s approval, parental consent was obtained beforehand for all the children. The study was carried out according to the ethical standards and requirements established by the Italian psychological association and by the local ethical committee of our University.

### Tasks

#### Domain‐specific abilities

##### Geometrical mental imagery test

This test, included in the Italian ‘*Geometria* *Test’* battery (Mammarella et al., 2012; Cronbach's α = .72), consists of twenty‐four tables divided into six different exercises. Each exercise requires to compose and decompose figures, find the solid figure corresponding to a plane representation of it, identify the volume of different figures by counting the cubes that make them up, identify an embedded figure, and colour the intersection between different figures. On average, this test takes about 20–30 min. The total number of exercises correctly solved was used for the analysis.

##### Geometrical declarative knowledge

This test, also included in the ‘Geometria Test’ battery (Mammarella et al., 2012), consists of 8 multiple choice questions that investigate the knowledge of geometrical‐related vocabulary (e.g., what is a segment?) and the names and properties of figures (e.g., which of these is a concave figure?). On average, this test takes about 10 min. Despite the fact that the test examines different aspects of geometrical declarative knowledge, as also confirmed by its relatively low consistency (α = .46), due to its limited number of items, only the total number of correct answers was used for the analysis.

##### Calculation

Calculation ability was assessed using the AC‐FL test (Caviola, Gerotto, Lucangeli, & Mammarella, 2016). This task consisted of three parts requiring to solve twenty‐four complex additions, subtractions, and multiplications, respectively (αs were .87, .86, and .79, respectively). Problems were presented in column, on three pages separately. Children had 2 min per page to solve as many problems as possible. The total number of problems solved in the three parts was used for the analysis.

##### Trapezoid problem‐solving tasks

**Figure 1:**
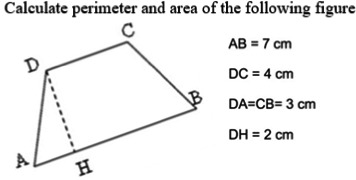
Example item for the non‐verbal geometric problem‐solving task.

An ad hoc measure was created to specifically assess children's competence in calculating the area or the perimeter of trapezoids. This task was created with the support of an expert in teaching geometry. Two parallel forms (A and B) were created to assess children immediately after the lecture and two weeks later. The two forms were validated for the current study showing good psychometric properties (*α*s were .74 and .82, respectively). The tasks consisted of a series of filler items and of eight items specifically concerning the trapezoid. Four items included some verbal descriptions (i.e., ‘July is drawing on the blackboard a rectangular trapezoid. Given that its major basis is 22 cm long, the minor basis is half long, the height is 9 and the oblique side is 14, calculate the perimeter’). The other four were based on the visual presentation of a trapezoid. For example, the child was shown the figure of an inclined isosceles trapezoid and the data necessary for calculating both the perimeter and the area, indicated with reference to the extremities of the sides (Figure 1). The score attributed for each item was: 1 point for the correct solution; 0.5 points if the procedure was correct but the calculation was wrong; 0 points in the other cases (for the overall score: min 0, max 8).

#### Domain‐general abilities – WM tasks

Participants were presented with six computerised tasks using E‐Prime software (Psychology Software Tools, Pittsburgh, PA). Three for V‐WM and three of VS‐WM. The partial credit score was used for scoring purposes (Allen, Giofrè, Higgins, & Adams, 2020; Giofrè & Mammarella, 2014) and was used for calculating the overall score for working memory tasks.

##### Visuospatial span – forward and backward

In the tasks, adapted from the classical Corsi (1972) tasks, children had to memorize the positions of series of black cells that appeared briefly, one at the time (1 s), in a 5 × 5 greed visible in the middle of the screen. At the end of a series, they had to indicate with the mouse the locations where they had previously seen the black cells taking into the account the order in which they had appeared. There were two different conditions, in which the children had to recall the cells in the forward and in the backward order respectively. The number of cells presented in black in each series ranged from two to eight. There was no time limit for recalling the cells, and the score was the number of cells accurately recalled in the right order (min 0, max 70, with αs of .77 and .76, respectively, for the forward and backward version).

##### Dot‐matrix task

This task (adapted from Miyake, Friedman, Rettinger, Shah, & Hegarty, 2001) assesses the active ability to simultaneously maintain and manipulate different spatial information. Children were presented with series of matrix equations to verify, each followed by a 5 × 5 grid containing a dot. In the matrix equations, children saw two successively presented segments and had to verify whether a third presented segment pattern correctly described the sum (or subtraction) of the previous equation matrix. At the end of each series of equations, children had to indicate with the mouse the positions where they had previously seen the dots. The sets of matrices were presented in four series of increasing length so that the location of 2–5 dots had to be remembered. The score corresponded to the number of dot locations correctly recalled (min 0, max 28; α = .69).

##### Forward and backward word span

In these tasks, adapted from the classical digit span tasks (Wechsler, 2004), but using words rather than digits (Giofrè, Donolato, & Mammarella, 2018), lists of words were presented verbally at a rate of 1 item per second and children had to recall them in the forward or backward order, respectively. The lists were presented proceeding from the shortest (two items) to the longest (eight items) lists. We considered the number of words accurately recalled in the correct order (min 0, max 70, αs of .71 and .71, respectively, for the forward and backward version of the span).

##### Listening span test (LST)

In the task, representing the Italian version (Palladino, 2005) of the task proposed by Daneman and Carpenter (1980), children listened to series of sentences including from two to five sentences. After listening each sentence, the children had to judge whether the sentence was true or false. Then, at the end of each series of sentences, they had to recall the last word of each sentence in order of presentation. The score was the number of words accurately recalled (min 0, max 28; α = .58).

#### Instructions to teachers and lecture on the trapezoid

**Figure 2:**
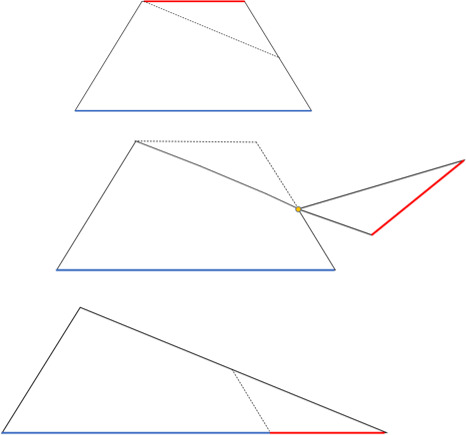
Trapezoid card: how to decompose a trapezoid in a triangle.

Teachers who had accepted to participate to the project were contacted at the beginning of the school year and accepted to follow the instructions and to avoid any anticipation on trapezoid before the lecture on trapezoid. They also controlled, at the beginning of the lecture, that all their pupils had no specific information on trapezoid. As far as the instructions used to explain the trapezoid, all teachers followed the same detailed script in order to ensure a similar lesson in every class and to control as much as possible for teaching method and quality. The lecture, lasting about 60min, involved both direct and strategic instructions as in CSA (Cass et al., 2003). The overall instructions on how to calculate the area of the trapezoid entangled the decomposition of the trapezoid into a triangle (Figure 2). The lecture included an introduction on the different trapezoid types, names and proprieties and information on how to calculate the perimeter (direct instruction). After this phase, the teacher started explaining the formula to calculate the trapezoid’s area by means of CSA teaching sequence. Every teacher was provided with a trapezoid card (manipulative) that could be easily decomposed to form a triangle with the length’s base corresponding to the sum of both trapezoid bases (Figure 2). Starting by showing this trapezoid model, the teacher could visually demonstrate that the overall area of the trapezoid is equivalent to that of a triangle whose base is the sum of the two trapezoid’s bases. Thus, children understood that the trapezoid area may be easily obtained from a small adjustment of the well‐known formula of the triangle area. In explaining this, children were asked to build their own trapezoid, to decompose it in two parts and then rearrange them in a triangle. These concrete instructions allowed pupils to have a more practical and comprehensive knowledge throughout a direct manipulation of a tangible geometric figure. In the final part of the lecture, the instruction returned to involve mainly abstract information, and the teacher explained formulas and basic concepts concerning trapezoid.

### Procedure

Participants were tested over the course of four both group and individual sessions. In the first session, the whole class was tested using the following tasks: calculation, declarative knowledge, and geometrical mental imagery. In the second session, children were individually tested with the six computerised WM tasks. Tasks were counterbalanced, using the Latin square, to reduce practice effects. The third session took place soon after the lecture on the trapezoid, while the fourth session took place two weeks later. During these two last sessions, children were administered the two parallel versions of the geometric trapezoid tasks. Teachers were instructed to avoid any anticipation concerning trapezoid before the lesson and not propose any exercise on trapezoid during the two‐week gap between sessions.

## Results

### Preliminary analyses

An exploratory factor analysis, using principal axis factoring as extraction method (PAF), with Promax rotation, was performed on WM tasks to verify whether these tasks could load on two latent factors, that is, verbal and visuospatial WM, respectively. The scree plot showed two factors with eigenvalues greater than 1.00, which explained the 41.6% and the 11.7% of variance respectively. The first factor explained the common variance for the six tasks, but also specific variance related to V‐WM. Only the forward and the backward word span test, alongside with the listening span test, substantially loaded on Factor 1, while the forward and the backward visuospatial span tests, alongside with the dot matrix, loaded on Factor 2 (Table 1).

**Table 1:** Factor loadings for the exploratory factor analysis concerning the six working memory tasks

	V‐WM	VS‐WM
Forward word span	.930	
Backward word span	.612	
Listening span test	.414	
Forward visuospatial span		.635
Backward visuospatial span		.795
Dot‐Matrix		.736

Note

Loadings < .35 have been omitted.

V‐WM = verbal working memory; VS‐WM = visuospatial working memory.

Descriptive statistics and correlations for all measures are shown in Table 2. The two factors (i.e., V‐WM and VS‐WM) were highly correlated (.605) and presented significant correlations with the score at the Trapezoid A task (respectively .406 and .475). In addition, all the variables individuated as potential predictors of geometrical learning had a significant, although not particularly high, correlation with the success in the trapezoid tasks. Correlations of the domain‐specific and the domain‐general tasks with the Trapezoid tasks ranged between .333 and .475, while the two tasks on the trapezoid were highly correlated (.634) suggesting that the outcomes obtained with the first test were substantially replicated at the follow‐up.

**Table 2:** Correlations and descriptive statistics of the predictors and the learning measures

	1	2	3	4	5	6	7
1 Geometrical mental imagery	—						
2 Geometric vocabulary	.438***	—					
3 Calculation	.272**	.141	—				
4 V‐WM	.349***	.245*	.351***	—			
5 VS‐WM	.325***	.232*	.326***	.605***	—		
6 Trapezoid problem A	.338***	.456***	.397***	.406***	.475***	—	
7 Trapezoid problem B	.333***	.408***	.431***	.395***	.369***	.634***	—
*M*	14.20	5.94	38.00	—	—	4.84	4.03
*SD*	4.12	1.60	10.4	—	—	2.12	2.57

Note

Factor scores for WM are standardized.

V‐WM = verbal working memory; VS‐WM = visuospatial working memory.

* *p* < .05.

** *p* < .01.

*** *p* < .001.

To verify whether the acquisition of the knowledge on the trapezoid remained stable over time, we performed a paired sample *t*‐test. The mean difference between Trapezoid problem A and B was significant, *M*_diff_ = 0.81; *t*(104) = 4.075, *p *< .001, but the effect size was not large (Cohen’s *d* = 0.447), meaning that there was a good maintenance of the information acquired during the lesson.

### Regression

**Figure 3:**
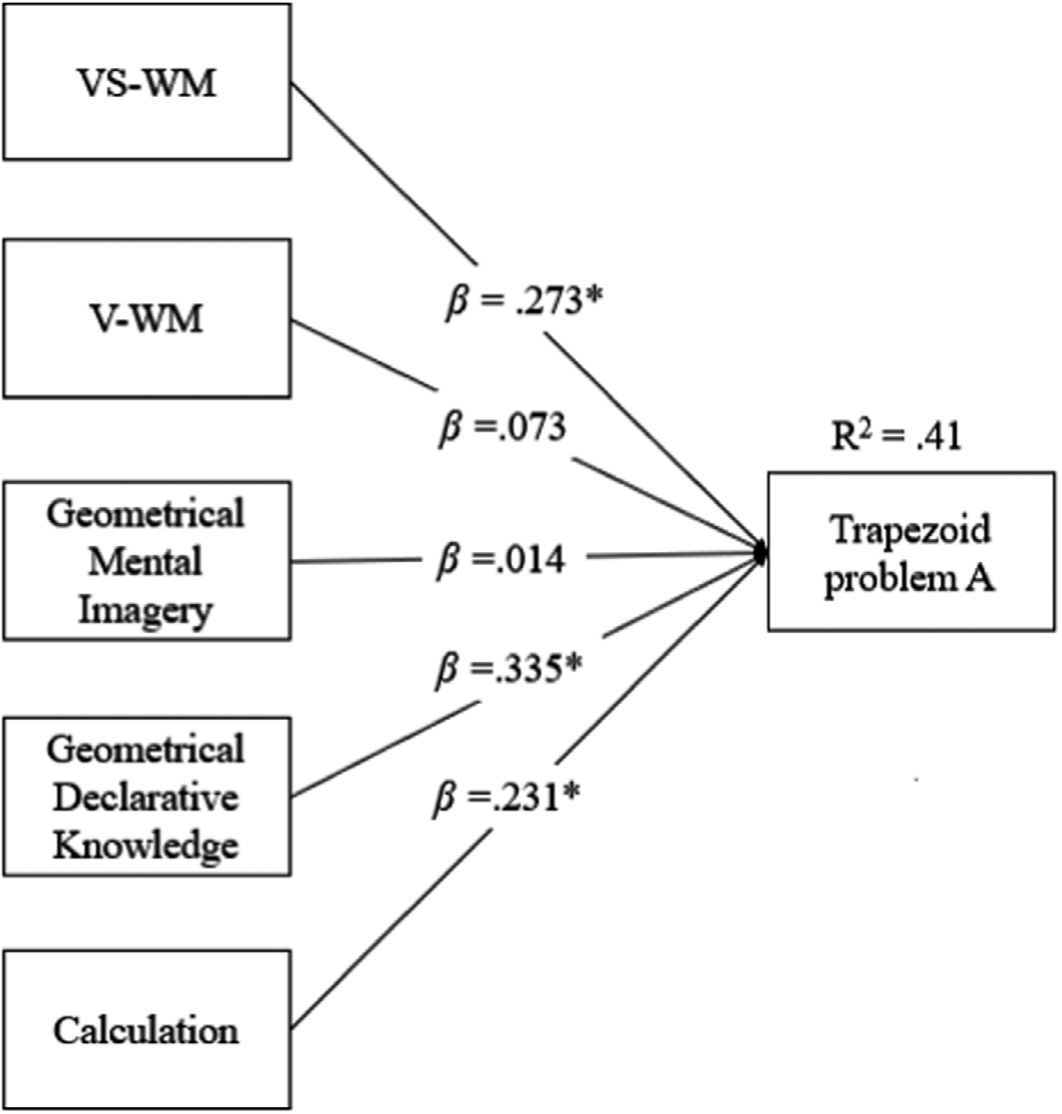
Regression model investigating the role of the different cognitive processes on the acquisition of the trapezoid geometrical concept (the significant contributions are indicated with an asterisk*). *Note*. VS‐WM = visuospatial working memory, V‐WM = verbal working memory. Loadings < .35 have been omitted.

A regression model was used to investigate the role of the different cognitive processes on the acquisition of the trapezoid geometrical concept. In particular, WM factorial scores (verbal and visuospatial), declarative knowledge, geometrical mental imagery, and calculation were entered as predictors, while the performance at the Trapezoid problem‐solving task administered at the end of the lesson was included as the outcome variable. The model was statistically significant and explained a relevant portion of the variance (*R* ^2^ = .41, *p *< .001). In this model, only VS‐WM (β = .273, *p* < .01), declarative knowledge (β = .335, *p* < .001), and calculation (β = .231, *p* < .01) were statistically significant, suggesting that the contribution of geometrical mental imagery and V‐WM was not unique, but was explained by the other three variables (Figure 3).

### Additional analyses

As some evidence indicated small, but statistically significant gender differences in mathematics tasks, including the geometric ones (see Giofrè, Cornoldi, Martini, & Toffalini, 2020 for a recent review of the issue; see also Zhang, 2020 for a discussion), we decided to verify the role of gender differences in the performance with the Trapezoid. A hierarchical regression was performed, including gender in the first step, while all the other predictors in a subsequent step. Results indicated that the effect of gender was not statistically significant and small in terms of magnitude (*R* ^2^ = .027, *p* = .095), thus overall confirming that gender differences, if any, tended to be rather small. Importantly, the results observed in the regression above changed only slightly when gender was entered into the regression. The final model including all the predictors was statistically significant and explained the 41% of the variance (Δ*R* ^2^ = .382, *p *< .001). Consistently with our first regression, VS‐WM (β = .273, *p *< .01), declarative knowledge (β = .336, *p* < .001), and calculation (β = .231, *p* < .01) were the only predictors to be statistically significant.

## Discussion

The present study aimed to examine the role of WM and of domain‐specific variables on the acquisition of advanced geometrical knowledge, based on the exemplar case represented by the learning of a new geometric figure (the trapezoid) in Y4 children. We focused on this particular group because in Year 4 the geometric curriculum becomes more formal and children begin to learn how to calculate area and perimeter of complex figures. We considered the trapezoid because this is one of the most complex and cognitively demanding concepts, and the level of children’s familiarity with this figure is typically low and can be controlled by asking to the teachers to avoid any reference to the geometry of trapezoids.

To examine factors affecting the acquisition of trapezoid knowledge, we implemented a CSA teaching sequence, including frontal lecturing, but also requiring the active manipulation of geometric figures. Previous studies highlighted the importance to promote learning sessions based on interactive and manipulatives activities, in which children can actively construct knowledge (Vygotsky, 1978). Children included in the present report achieved an acceptable performance (60.5%) after a single one‐hour lecture on the trapezoid. This suggests that the lecture was effective in fostering children performance in geometric competence. Furthermore, at the follow‐up, children’s geometrical performance only slightly decreased (with a high correlation between the two measures), as students were able on average to solve about half of the problems (50.38%), despite the fact that two weeks had passed from the first assessment without providing any further exercise or guidance on this topic.

Our main purpose was to investigate the role of different specific‐ and general‐domain abilities in the online processes of acquisition of the trapezoid. To this purpose, we had assessed geometrical mental imagery, declarative knowledge, calculation, and visuospatial and verbal WM. Our results indicate that VS‐WM was explaining a portion of the variance over and above the effect of the domain‐specific predictors and of V‐WM. On the contrary, V‐WM, despite its significant correlation with the trapezoid task, did not enter into the regression. This result was probably due to the fact that part of the variance of V‐WM was shared by VS‐WM, but also to the fact that geometry largely relies on visual processes and this was emphasized by the specific features of the lecture on the trapezoid, which was involving the visuospatial manipulation of the stimuli (Allen & Giofrè, 2020). In particular, the lecture included the decomposition of the trapezoid into smaller pieces (i.e., triangles). Therefore, a good VS‐WM capacity could probably facilitate children to perform visuospatial manipulations, helping them in the resolution of a problem involving a complex figure. As for gender effects, differences in geometry were also investigated. Gender was not a statistically significant predictor of the performance in the trapezoid, with effects small in terms of magnitude, which confirms the observation that gender differences in mathematics, tend to be small in terms of magnitude, as Giofrè et al. (2020) observed with a sample of over 13 million Italian children.

We could speculate that different teaching methods and approaches could influence the cognitive demands of a particular task. The current literature is scarce and conflicting results have been found on the verbal versus visuospatial WM involvement in geometric tasks (Peng, Namkung, Barnes, & Sun, 2016). Most studies have found a significant impact of VS‐WM (Allen & Giofrè, 2020; Giofrè et al., 2013) but other reports (Giofrè et al., 2014) have stressed the importance of V‐WM. In a traditional lecture involving theorems or merely calculus, children are probably relying heavily on some verbal aspect of the problem, such as vocabulary and V‐WM (Giofrè et al., 2014). However, if the teaching method specifically requires the manipulation of visuospatial materials, as in this case, then other cognitive mechanisms (e.g., VS‐WM) may play a stronger role. Such a finding is very intriguing because suggests that the teaching method could somehow interact with the cognitive abilities of the students receiving the lecture, and this could have important implications for the educational setting.

As for the other variables included in the current report, our results indicate that calculation, and geometrical declarative knowledge were significantly predictors of geometrical learning. This result is particularly important, as it highlights the importance of both procedural and declarative knowledge in solving geometric problem‐solving tasks (Rittle‐Johnson & Alibali, 1999; Rittle‐Johnson, Siegler, & Alibali, 2001). Declarative knowledge (i.e., knowledge of geometrical concepts, including technical terms and geometrical principles) supports the understanding of the problem and the selection of the correct procedure to solve it, while procedural knowledge (e.g., calculation) enables the correct application of the selected procedure. Notably, our results showed that the relationship between geometrical declarative knowledge and geometrical learning of the trapezoid was higher in terms of magnitude as compared to calculation abilities. This confirms that, at least for Y4, the acquisition of the geometric terminology and basic concepts, which tend to be rather specific, have a predominant role.

Concerning geometrical mental imagery, previous studies showed a relationship between this ability and geometry, suggesting that the ability to generate and manipulate mental representation of geometric figures is predictive of geometrical learning (Bizzaro et al., 2018; Weckbacher & Okamoto, 2014). Conversely, in our study we found that the impact of geometrical mental imagery on geometrical learning was negligible when this task was included in association with other cognitive predictors. Such a finding could be due to the fact that mental imagery is in part overlapping with the other predictors, as it has in particular been suggested for VS‐WM (Albers, Kok, Toni, Dijkerman, & De Lange, 2013; Cornoldi & Vecchi, 2003; Tong, 2013) that supports the ability to actively represent and manipulate visual information.

The present study has some important educational implications for teaching advanced geometrical concepts in children with or without learning disabilities. An in‐depth knowledge of factors affecting learning could help teachers to improve their teaching methods and to better understand how to help students having specific difficulties with this area (Bizzaro et al., 2018). Our results stress the role of VS‐WM in learning geometry, highlighting the importance to support and promote an adequate development of this ability and to consider the role of this variable early in the curriculum. These findings provide evidence that geometrical learning, involving the ability of calculating the area and the perimeter of a new figure, relies on other geometrical concepts (e.g., points, lines, or diagonals; see Fisher et al., 2013). These results also corroborate the efficacy of teaching strategies, such as the CSA, for teaching complex geometrical concepts integrating the traditional lecture with manipulatives materials. We believe that this could play an important role not only with primary school children with or without learning difficulties, but also later in the curriculum.

It is worth noting that even though teachers were explicitly asked to not provide any information about the trapezoid before the lesson and during the interval between test and follow‐up, we cannot rule out the possibility that children involved in the study might have had some previous knowledge about the trapezoid, or they might have been informally exposed to this figure within the family setting. Future studies should therefore address this issue by including a baseline assessment of children’s intuitive knowledge of the trapezoid before being formally introduced to this figure within the school setting.

Our study has a series of other limitations. First, we only had limited access to the school setting and our agreement with the schools did not allow for having a more complete assessment nor a follow‐up after a longer period of time. Regarding the cognitive processes implicated in learning geometry, the portion of explained variance was statistically significant, but not particularly large in terms of the effect size. Other domain general factors could affect geometrical learning (Giofrè et al., 2014) and should be considered in future studies, for example, arithmetic abilities other than calculations, more general mathematical problem‐solving abilities (LeFevre et al., 2010), or metacognitive and motivational aspects such as self‐efficacy and math anxiety (Aydın & Ubuz, 2010; Donolato, Toffalini, Giofrè, Caviola, & Mammarella, 2020; Justicia‐Galiano, Martín‐Puga, Linares, & Pelegrina, 2017; Mammarella, Caviola, Giofrè, & Borella, 2018), that presumably affect geometrical learning as they affect other aspects of mathematical learning. In addition, other factors such as student–teacher relationship or the teaching quality might have an impact on this important acquisition and should be considered in future studies involving a higher number of classes and teachers (see Semeraro, Giofrè, Coppola, Lucangeli, & Cassibba, 2020 on this point).

In conclusion, our study highlights the importance of VS‐WM, geometrical declarative knowledge, and calculation skills in solving problems on a new and particularly difficult geometrical concept for primary school children. Our results could help teachers to understand the processes and abilities underpinning geometrical learning, a subject that is very complex and articulated, and has unfortunately received until now insufficient attention by the current literature.

## Conflicts of interest

All authors declare no conflict of interest.

## Author contributions

Sara Caviola (Conceptualization; Data curation; Methodology; Writing – original draft; Writing – review & editing) David Giofrè (Data curation; Formal analysis; Writing – original draft; Writing – review & editing) Carlotta Rvella (Conceptualization; Data curation; Project administration; Writing – original draft) Cesare Cornoldi (Conceptualization; Supervision; Writing – original draft).

## Data Availability

Our agreement with schools involved in the current study did not include the possibility of sharing the data for the current report. However, most of the analyses, regressions *t*‐test and regressions here reported, can be easily derived by using the information included in the tables.
